# Testing for myelin oligodendrocyte glycoprotein antibodies: Who, what, where, when, why, and how

**DOI:** 10.1177/13524585251313744

**Published:** 2025-01-24

**Authors:** Adrian Budhram, Eoin P Flanagan

**Affiliations:** Department of Clinical Neurological Sciences, Western University, London Health Sciences Centre, London, ON, Canada; Department of Pathology and Laboratory Medicine, Western University, London Health Sciences Centre, London, ON, Canada; Department of Neurology, Mayo Clinic, Rochester, MN, USA; Department of Laboratory Medicine and Pathology, Mayo Clinic, Rochester, MN, USA

**Keywords:** MOG, MOGAD, demyelinating disease, neural antibody, autoantibody

## Abstract

Testing for myelin oligodendrocyte glycoprotein immunoglobulin G antibodies (MOG-IgG) is essential to the diagnosis of MOG antibody-associated disease (MOGAD). Due to its central role in the evaluation of suspected inflammatory demyelinating disease, the last 5 years has been marked by an abundance of research into MOG-IgG testing ranging from appropriate patient selection, to assay performance, to utility of serum titers as well as cerebrospinal fluid (CSF) testing. In this review, we synthesize current knowledge pertaining to the “who, what, where, when, why, and how” of MOG-IgG testing, with the aim of facilitating accurate MOGAD diagnosis in clinical practice.

## Introduction

**Figure 1.**
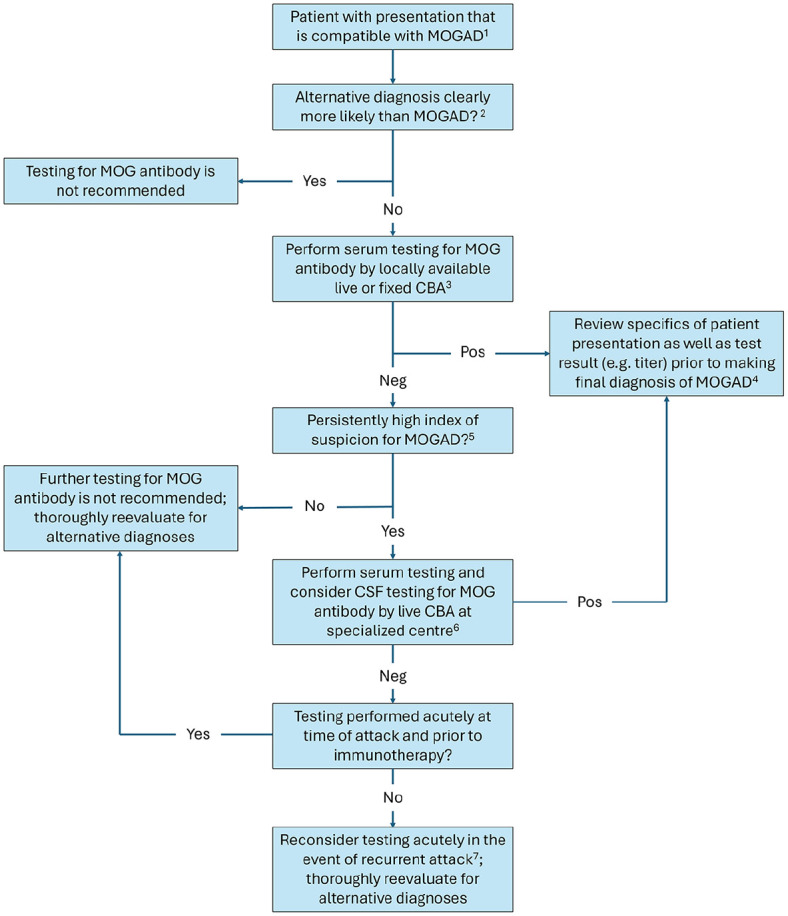
Proposed approach to MOG antibody testing in clinical practice. ^1^Spectrum of presentations compatible with MOGAD is subject to evolution over time, as evidenced by recently described presentations that are potentially suggestive of MOGAD yet not included in the 2023 criteria (see text). ^2^For a given patient, there may be competing diagnostic considerations (e.g. MOGAD vs. MS and MOGAD vs. infectious encephalitis) but no alternative diagnosis that is clearly more likely than MOGAD, despite completion and review of relevant investigations (e.g. neuroimaging, testing for CSF-specific oligoclonal bands, and testing for CSF viral polymerase chain reactions (PCRs)); in such cases with persistent diagnostic uncertainty, MOG-IgG testing may appropriately be pursued to aid in diagnostic clarification. However, in cases where an alternative diagnosis is clearly more likely than MOGAD (e.g. adult with characteristic neuroimaging for MS and CSF-specific oligoclonal bands, characteristic neuroimaging for HSV encephalitis and positive CSF HSV-1 PCR), testing for MOG-IgG is not recommended due to the high proportion of false-positive results when testing is performed in such low-probability scenarios. ^3^For some clinicians, live cell-based assay CBA performed at a specialized center may be locally available and thus appropriately serve as first-line testing. However, local availability of MOG-IgG assays is subject to practical considerations such as test cost and turnaround time in addition to diagnostic performance; this proposed approach acknowledges that routine testing of samples by live CBA performed at a specialized center may not be possible for many clinicians worldwide. ^4^In patients with a positive MOG-IgG result, the specifics of the patient presentation (e.g. clinical symptoms, neuroimaging findings as well as their evolution over time, and ancillary laboratory test findings such as CSF-specific oligoclonal bands) as well the antibody result (e.g. titer) should be reviewed prior to assigning a final diagnosis of MOGAD. It should be reaffirmed that the patient has a presentation compatible with MOGAD and no more likely alternative diagnosis, recognizing that the clinical impression may change between ordering and receiving a MOG-IgG test result due to new clinical or ancillary test findings that become available in the interim; if new information suggests that the patient presentation is not compatible with MOGAD and/or that there is a more likely alternative diagnosis, then the diagnosis of MOGAD should be critically re-evaluated despite having a positive antibody result. Knowledge of MOG-IgG titers with application of the 2023 criteria can aid in the diagnosis of MOGAD, recognizing that the need for supportive features in patients with low serum MOG-IgG titers improves specificity of the criteria but may come at some cost to sensitivity (see text). ^5^What constitutes a persistently high index of suspicion for MOGAD is subject to clinical judgment, but concern may be particularly high in certain scenarios (e.g. patients with highly characteristic presentations or recurrent attacks compatible with MOGAD) despite negative MOG-IgG results by locally available assays. Restricting send-out testing to such scenarios balances practical limitations (e.g. cost and turnaround time) with likelihood of benefit. ^6^Live CBA at a specialized center refers to testing performed at a laboratory with expertise in this methodology and published assay performance data confirming high diagnostic accuracy. In addition to serum, submission of CSF for MOG-IgG testing may also be considered given the persistent suspicion for MOGAD, although isolated CSF MOG-IgG positivity should be scrutinized (see text). ^7^If testing was performed close to time of symptom onset but after administration of acute immunotherapy then repeat testing at least 3 months later may be considered even in the absence of a recurrent attack, recognizing that data supporting this practice are more limited. CBA: cell-based assay; MOG: myelin oligodendrocyte glycoprotein; MOGAD: MOG antibody-associated disease.

Detection of myelin oligodendrocyte glycoprotein immunoglobulin G antibodies (MOG-IgG) is central to the diagnosis of MOG antibody-associated disease (MOGAD).^1^ Increasing awareness of patient presentations compatible with MOGAD has spurred intense investigation into all facets of MOG-IgG testing, from appropriate patient selection, to assay performance, to utility of serum titers as well as cerebrospinal fluid (CSF) testing. We herein synthesize current knowledge pertaining to the “who, what, where, when, why, and how” of MOG-IgG testing for the practicing clinician and propose a testing approach that may be applied broadly (Figure 1). Testing for immunoglobulins other than IgG, while of recent interest,^2^ is not routinely clinically available and thus not discussed further.

## Who to test

### Testing should be restricted to patients with compatible presentations

In 2018, international recommendations suggested restricting MOG-IgG testing to patients with clinical and paraclinical findings that were typical of MOGAD and/or atypical for multiple sclerosis (MS).^3^ At this time, established acute demyelinating syndromes compatible with MOGAD included optic neuritis, myelitis, brainstem demyelination, and acute disseminated encephalomyelitis (ADEM). The more recent 2023 MOGAD criteria similarly emphasized the importance of restricting testing to patients with compatible presentations and highlighted “core clinical demyelinating events” consisting of optic neuritis, myelitis, brainstem/cerebellar demyelination, ADEM, cerebral monofocal or polyfocal deficits, and cerebral cortical encephalitis (CCE).^1^ The inclusion of CCE in the 2023 criteria, a presentation not recognized in the 2018 recommendations due to its more recent characterization, reflects an evolution in our understanding of which presentations may be suspicious for MOGAD. The ongoing nature of this evolution is evidenced by presentations not included in the 2023 criteria but recently identified as potentially compatible with MOGAD, such as aseptic meningitis.^4^ It has also recently been highlighted that children with MOG-IgG positivity may present with cerebral syndromes, pleocytosis, and normal brain magnetic resonance imaging (MRI) not clearly explained by radiologic lag, possibly indicating another presentation of MOGAD not captured by the 2023 criteria.^5,6^ Future criteria will be tasked with the responsibility of incorporating novel presentations while discouraging indiscriminate MOG-IgG testing in mimicking disorders (e.g. infectious meningitis) to minimize risk of misdiagnosis.

### Testing of low-probability patients reduces positive predictive value

It is intuitive that a diagnostic test should only be ordered in patients for whom the disease of interest is suspected. Nonetheless, particular emphasis has been placed on appropriate patient selection for MOG-IgG testing to preserve its positive predictive value (PPV), which refers to the proportion of MOG-IgG-positive patients who actually have MOGAD. The reason for this emphasis is that MOG-IgG assays have high but imperfect specificity, which can translate to significant lowering of PPV when testing is performed in populations with a low prevalence of MOGAD. As an illustrative example, consider the diagnostic performance of aquaporin-4 (AQP4)-IgG and MOG-IgG live cell-based assays (CBAs) offered by the Mayo Clinic.^7,8^ The absolute difference in the specificities of these two tests is reportedly small (specificity of ≈100% for AQP4-IgG vs. 97.8% for MOG-IgG, difference of approximately 2%); however, this small difference in specificities translates to a large difference in PPVs in clinical practice, where testing may be performed in low-probability patients (PPV of ≈100% for AQP4-IgG vs. 72% for MOG-IgG, difference of nearly 30%). Restricting MOG-IgG testing to patients with reasonable suspicion for MOGAD (i.e. compatible presentation and no alternative diagnosis that is clearly more likely, see Figure 1) is, therefore, essential to reducing the proportion of false-positives that may contribute to misdiagnosis.

## How and where to test

### CBAs are the recommended test methodology

Early studies of MOG-IgG, which relied on assays such as immunoblot or enzyme-linked immunosorbent assay (ELISA) that detect antibodies against denatured protein, focused on its potential relevance in MS and reported conflicting findings.^9^ High rates of MOG-IgG positivity among healthy controls using these assays cast further doubt on its relevance to inflammatory demyelinating disease.^9^ It was not until the use of conformational MOG,^10^ followed thereafter by widespread availability of CBAs expressing conformational MOG, that MOGAD emerged as an entity with distinct clinical, neuroimaging and pathologic features. Investigations into serum MOG-IgG testing by CBA have reported high (⩾98%) specificity across assays, supporting the recommendation for their use in the 2023 criteria.^11^ Use of an IgG Fc or IgG1-specific secondary antibody is recommended for MOG-IgG detection by CBA, although the use of an IgG (heavy and light) secondary antibody with externally validated in-house assays is also considered acceptable.^1^ Importantly, ELISA is not recommended for MOG-IgG detection due to its inferior diagnostic performance when compared to CBA.^12^ For laboratories that utilize murine tissue-based assays for antibody testing in autoimmune encephalitis it is important to recognize that up to 13% of patients with CSF MOG-IgG positivity can have white matter immunostaining on CSF testing,^13^ although CBA remains the recommended methodology for MOG-IgG detection due to its higher sensitivity and specificity.

### Live versus fixed CBAs for antibody detection

Live CBA testing, which has been referred to as the “gold standard” for MOG-IgG detection, is generally restricted to specialized laboratories due to its resource-intensive nature. Meanwhile, fixed CBAs, whereby fixation allows for assay transport and commercialization, are more easily implemented in many clinical laboratories and are thus in widespread use. While fixation facilitates assay distribution it may result in loss of some conformational epitopes of MOG which could adversely impact sensitivity, underscoring the need for comparison studies to live CBA.^11,12^ A multicenter study compared the diagnostic performance of live versus fixed MOG-IgG CBAs.^12^ Among samples that were classified as clearly positive (*N* = 39) or negative (*N* = 40) for MOG-IgG by previous live CBA testing, overall agreement across the seven live CBAs included in this study was excellent at 96%.^12^ Agreement fell to 90% when the one fixed CBA in this study was included in this analysis; this was driven by 4/39 samples (10%) that were positive by all seven live CBAs but discordantly negative by only the fixed CBA, suggesting that fixed CBA may miss 10% of clearly positive MOG-IgG results by live CBA.^12^ This study also examined samples that were classified as low/borderline-positive (N = 39) or borderline-negative (N = 13) for MOG-IgG by previous live CBA testing and found overall assay agreement to be significantly lower at 44%. Interestingly, among nine of these samples that were positive by seven CBAs but discordantly negative by only one CBA, the discordant assay was a live CBA in each case rather than the fixed CBA; all but one of these discordantly negative results (8/9, 89%) were generated by just two of the seven live CBAs included in this study. This suggests that, while live CBA may have overall higher sensitivity than fixed CBA, there are positive MOG-IgG results detected by fixed CBA that are missed by some live CBAs. In addition to variable sensitivity across in-house live CBAs, there may also be variable specificity; this is reflected by MOG-IgG positivity rates by live CBA in MS that range from 0.3% to as high as 9.9% exceptionally in one study, acknowledging that these differences may relate in part to how patients with MS are selected across studies and not solely assay performance.^14,15^ Taken together, these findings highlight the value of MOG-IgG live CBA performed at specialized centers that have expertise in this methodology and published assay performance data confirming high diagnostic accuracy.

Limitations to current studies of live versus fixed CBA for MOG-IgG detection include selection of samples that previously tested positive by live CBA (sampling bias),^12,16^ as well as a relative paucity of investigation into the potential for, and clinical significance of, samples that exhibit positivity for MOG-IgG by fixed CBA but not live CBA.^17^ Nonetheless, given reports that fixed CBA may miss 10%–15% of samples that test positive for MOG-IgG by live CBA performed at specialized centers,^12,16^ coupled with the potential for variable diagnostic performance across locally developed live CBAs, it would seem reasonable to arrange for send-out live CBA testing by a specialized center when there remains a high index of clinical suspicion (e.g. highly characteristic presentation or recurrent attacks compatible with MOGAD) despite negative serum results by locally available live or fixed CBA (Figure 1). In this scenario, CSF submission for MOG-IgG testing may be considered in addition to serum due to persistent suspicion for MOGAD (Figure 1), recognizing the need for cautious interpretation in cases of isolated CSF positivity (discussed later on). This stepwise approach balances the practicalities of test availability locally, which may not include live CBA performed at a specialized center due to considerations such as test cost and turnaround time in addition to diagnostic performance,^1^ with the potential benefit of send-out testing for patients in whom there is particular concern for MOGAD despite negative antibody results by locally available assays.

### Performing antibody titers can help inform likelihood of a false-positive result

Studies using both live and fixed CBA have found that low serum MOG-IgG titers have lower PPV for MOGAD, reflecting their lower disease specificity.^8,18,19^ This is well-illustrated by PPV data published by the Mayo Clinic, which found that serum titers of 1:20–40 had a PPV of only 51% while serum titers of 1:1000 had a PPV of 100%.^8^ Determining the titer of positive MOG-IgG results in patients with equivocal presentations and/or competing diagnostic considerations can thus help inform the likelihood of a false-positive result. To offset the lower specificity of low serum MOG-IgG titers, the 2023 criteria require the presence of additional supportive features prior to making a diagnosis of MOGAD.^1^ This requirement serves to increase the specificity of the 2023 criteria but may come at some cost to sensitivity, particularly in patients with incomplete investigations (e.g. lack of fundoscopy or orbital MRI with fat-saturated sequences in those with optic neuritis), delayed testing, or testing by fixed CBA (for which lower titers correlate with higher titers by live CBA);^16,17,20,21^ these factors should, therefore, be considered in patients with low serum titers who do not meet the 2023 criteria despite a high index of suspicion for MOGAD.

## What to test

### Serum is the preferred fluid to test; isolated CSF positivity requires cautious interpretation

The clinical manifestations of MOGAD have been characterized largely through examination of cohorts with MOG-IgG detected in serum, thereby establishing serum positivity as a valuable diagnostic biomarker. Early study of serum versus CSF MOG-IgG testing reported CSF positivity in only two-thirds of seropositive patients, suggesting higher sensitivity of serum testing.^22^ Yet subsequent work found that, while overall sensitivity of serum MOG-IgG testing was higher than CSF, isolated CSF MOG-IgG positivity occurred in 3/44 (7%) with non-MS inflammatory demyelinating disease.^23^ This raised the question of whether a subset of patients with MOGAD may harbor antibodies that are only detectable in CSF and motivated investigation into the utility of CSF MOG-IgG testing. While studies have generally corroborated that overall sensitivity of serum MOG-IgG testing is higher than that of CSF, the reported rates of isolated CSF MOG-IgG positivity in patients with presentations suggestive of MOGAD range substantially from 0% to 30%.^13,23–27^ Notably, CSF MOG-IgG testing by fixed CBA does not appear to provide additional sensitivity over serum testing, indicating that any potential benefit of CSF testing is limited to live CBA.^26^ Among patients with isolated CSF MOG-IgG positivity that have been reported in the literature, the proportion classified as false-positive similarly ranges widely from 0% to more than 50%.^24,25,27,28^ The significant variability in both yield and specificity of CSF MOG-IgG testing likely relates at least in part to differences in test implementation across centers, which can vary in terms of in-house assay parameters, secondary antibody used, and CSF testing dilutions.^25^ Review of the available data suggests that CSF MOG-IgG testing in seronegative patients should be restricted to those with a high index of suspicion for MOGAD. In similar fashion to low serum antibody titers, cases of isolated CSF MOG-IgG positivity should be scrutinized; in such patients, contacting the testing laboratory to review assay-specific performance data can help inform the likelihood of a true-positive result.

## Why and when to test

### Testing is performed primarily for diagnostic purposes

The central importance of MOG-IgG testing to the diagnosis of MOGAD is reflected not only by its nomenclature but also by its 2023 criteria, which require a positive MOG-IgG result by CBA.^1^ For this reason, MOG-IgG testing is performed primarily to confirm a diagnosis in suspected cases of MOGAD. Importantly, however, a diagnosis of MOGAD cannot be made solely based on a positive MOG-IgG result, as it also requires a compatible presentation and exclusion of alternative diagnoses.^1^ Routine testing of MOG-IgG to “rule out” MOGAD in patients with atypical presentations and/or more likely alternative diagnoses is, therefore, discouraged; in such patients, a positive MOG-IgG result does not establish a diagnosis of MOGAD and may contribute to delayed diagnosis of other etiologies for their presentation.

### Testing is ideally performed close to time of symptom onset and prior to immunotherapy

MOG-IgG levels are dynamic; they are generally highest at the time of attack and decline thereafter, with longer time from symptom onset to testing being associated with lower antibody titers.^19,21,29,30^ Testing performed after immunotherapy has also been associated with lower antibody titers.^19^ Taken together, these findings suggest that MOG-IgG testing is ideally performed close to time of symptom onset and prior to immunotherapy. However, this is not always feasible, particularly for patients who are assessed in the outpatient setting and thought to have had a remote attack suspicious for MOGAD. In such patients who are negative for MOG-IgG at time of assessment, clinicians should consider the possibility that antibody levels have fallen below the threshold of assay detection. The potential value of retesting for MOG-IgG if the patient has another attack suspicious for MOGAD in the future, at which time antibody levels may be higher, should also be kept in mind (Figure 1). In contrast, the yield of repeat testing in a patient with an initially negative MOG-IgG result acutely at time of presentation is estimated to be low at <2%.^31,32^ If testing was performed close to time of symptom onset but after administration of acute immunotherapy then repeat testing at least 3 months later may be considered even in the absence of a recurrent attack, recognizing that data supporting this practice are more limited.^1^

### Repeat testing to assess for persistent seropositivity may inform risk of relapse

While the diagnostic utility of MOG-IgG testing is established, the role of repeat testing in patients with MOGAD is less clear. Persistent seropositivity has been associated with a higher rate of relapse that ranges substantially in the literature from 24%–88%, likely reflecting heterogeneity in study design, patient demographics, patient presentation, assay used, treatments administered, and test timing.^28,31,33^ Longer time to antibody negativity has similarly been associated with relapsing disease, particularly among children.^28,34^ Although following serum antibody titers is generally not required in routine clinical practice, it is also noteworthy that higher titers at time of remission, but not at onset, have been associated with increased risk of relapse.^35^ While “persistent seropositivity” lacks a standardized definition, the pragmatic approach of repeating serum testing 6–12 months after disease onset to assess for antibody persistence is in alignment with the available literature. A positive MOG-IgG result at this juncture may suggest a higher risk of relapsing disease; however, given the variability in absolute relapse rates reported across studies employing different assays, the decision of whether to administer long-term immunotherapy following a single attack of MOGAD should be based on clinical trajectory and not solely on persistent seropositivity.

## Conclusion

MOG-IgG testing has revolutionized the assessment of patients with inflammatory demyelinating disease and led to the discovery of MOGAD as a discrete entity with unique clinical features, imaging findings, treatment considerations, and prognosis. As knowledge of “who, what, where, when, why, and how” to test for MOG-IgG rapidly advances, clinicians benefit from having a practical approach to this testing as well as a working understanding of its limitations to ensure accurate diagnosis. Future work is sure to further optimize testing algorithms for MOG antibody testing and refine the diagnostic approach to patients with suspected MOGAD.
